# The economic burden of cardiac implantable electronic device infections in Alberta, Canada: a population-based study using validated administrative data

**DOI:** 10.1186/s13756-023-01347-4

**Published:** 2023-12-05

**Authors:** Elissa Rennert-May, Derek Chew, Kristine Cannon, Zuying Zhang, Stephanie Smith, Teagan King, Derek V. Exner, Oscar E. Larios, Jenine Leal

**Affiliations:** 1 Department of Medicine, University of Calgary, Calgary, AB Canada; 2 Department of Community Health Sciences, University of Calgary, Calgary, AB Canada; 3 O’Brien Institute for Public Health, University of Calgary, Calgary, AB Canada; 4 Department of Microbiology, Immunology and Infectious Diseases, University of Calgary, Calgary, AB Canada; 5 Snyder Institute for Chronic Diseases, University of Calgary, Calgary, AB Canada; 6 Libin Cardiovascular Institute, University of Calgary, Calgary, AB Canada; 7 Department of Cardiac Sciences, University of Calgary, Calgary, AB Canada; 8 Infection Prevention and Control, Alberta Health Services, Calgary, AB Canada; 9 Department of Medicine, University of Alberta, Edmonton, AB Canada; 10 Department of Pathology and Laboratory Medicine, University of Calgary, Calgary, AB Canada

**Keywords:** Economic burden, Surgical site Infections, Healthcare utilization

## Abstract

**Background:**

Cardiac implantable electronic devices (CIED) are being inserted with increasing frequency. Severe surgical site infections (SSI) that occur after device implantation substantially impact patient morbidity and mortality and can result in multiple hospital admissions and repeat surgeries. It is important to understand the costs associated with these infections as well as healthcare utilization. Therefore, we conducted a population-based study in the province of Alberta, Canada to understand the economic burden of these infections.

**Methods:**

A cohort of adult patients in Alberta who had CIEDs inserted or generators replaced between January 1, 2011 and December 31, 2019 was used. A validated algorithm of *International Classification of Diseases* (ICD) codes to identify complex (deep/organ space) SSIs that occurred within the subsequent year was applied to the cohort. The overall mean 12-month inpatient and outpatient costs for the infection and non-infection groups were assessed. In order to control for variables that may influence costs, propensity score matching was completed and incremental costs between those with and without infection were calculated. As secondary outcomes, number of outpatient visits, hospitalizations and length of stay were assessed.

**Results:**

There were 26,049 procedures performed during our study period, of which 320 (1.23%) resulted in SSIs. In both unadjusted costs and propensity score matched costs the infection group was associated with increased costs. Overall mean cost was $145,312 in the infection group versus $34,264 in the non-infection group. The incremental difference in those with infection versus those without in the propensity score match was $90,620 (Standard deviation $190,185). Approximately 70% of costs were driven by inpatient hospitalizations. Inpatients hospitalizations, length of stay and outpatient visits were all increased in the infection group.

**Conclusions:**

CIED infections are associated with increased costs and are a burden to the healthcare system. This highlights a need to recognize increasing SSI rates and implement measures to minimize infection risk. Further studies should endeavor to apply this work to full economic evaluations to better understand and identify cost-effective infection mitigation strategies.

**Supplementary Information:**

The online version contains supplementary material available at 10.1186/s13756-023-01347-4.

## Introduction

Cardiac Implantable Electronic Devices (CIEDs) are being prescribed with increasing frequency due to expanding indications and advances in technology [1]. These devices are among the highest volume and most expensive device procedures in North America, therefore complications can be very costly [2–4] CIED surgical site infections (SSIs) are becoming increasingly prevalent and outpacing the rate of implantation of devices [1]. The rates of CIED SSIs vary from approximately 1–4% depending on a variety of factors including patient comorbidities and implanting centers [5, 6]. These infections can result in substantial negative impact to patient quality of life due to recurrent and lengthy hospital admissions, the need for additional procedures and prolonged courses of antibiotics [7]. Additonally, SSIs are very costly to the healthcare system, with some studies suggesting tens of thousands of dollars attributable to CIED infections in both Canada and the US [8, 9]. However, there is still a lack of high quality data to support this.

Prior studies assessing the economic burden of CIED infections have generally relied on the use of administrative data or insurer databases as the methods of tracking infection. However, these methods have not been adequately validated [8, 10, 11].

The primary objective of this study was to assess the attributable cost of CIED infections in a Canadian population that was identified using validated administrative data as a method to track infections. The secondary objectives of this work were to explore healthcare utilization associated with CIED infections including length of stay (LOS) in hospital, number of admissions, and outpatient visits.

## Methods

### Overview and study design

This was a population-based cohort study in Alberta, Canada, a province of ~ 4 million people with a single healthcare system, Alberta Health Services (AHS), that included all patients who underwent CIED implantation, including those who subsequently developed a SSI. Patients were identified using a centralized CIED database (Paceart™) and these data were linked to AHS health administrative data that captures patient comorbidities and healthcare costs.

### Patient cohort

We identified a cohort of adult patients (i.e., age ≥ 18 years) who underwent a new CIED implantation (including pacemaker (PM), implantable cardioverter defibrillator (ICD), or cardiac resynchronization therapy (CRT)) or generator replacement between January 1, 2011, and December 31, 2019. The infection group was identified using Discharge Abstract Database (DAD) and *International classification of Diseases 10th revision Canada* (ICD-10-CA) codes (T827, T857, I330, I339, I38, I398, L0330, L0339, L038, L039) that were previously validated as able to identify complex CIED SSIs (i.e. deep and organ space but not superficial SSIs) with sensitivity and specificity > 90% [12]. All infections that occurred within one year from the index date of CIED implantation were tracked. Patients who had their device implanted outside of Alberta were excluded from the study.

### Data sources

#### Paceart™

CIED implantations were identified using the Paceart™ database which contains all device-related clinical encounters for patients followed within the province of Alberta, Canada. Paceart™ contains information regarding indications for device implantation, type of device, date of operation and basic demographic information including sex. Repeat procedures within a two-year period from the index surgical date were censored. This avoided double counting patient encounters months later as only the initial implant was counted as an index procedural date.

#### AHS analytics

AHS analytics data which provides healthcare information on all Alberta residents with an Alberta Health Care Insurance Plan (> 99% of provincial coverage) was used. This data repository provided records from both DAD which was used for tracking infection cases as described above. Both DAD and the national ambulatory care reporting system (NACRS, which contains data for hospital based and community based ambulatory care including day surgery, outpatient and community ambulatory clinics and emergency department visits) were used to obtain demographic information about the patient cohort including comorbidities which were collected using a two-year retrospective review. Rural versus urban location and the Pampalon Deprivation Index was collected as well. The Pampalon Deprivation Index is a composite index using Canadian census data in order to create a measure of socioeconomic disparity, [13] and urban versus rural residence data was determined from patient postal codes.

A mix of gross costing and micro-costing was used to assess economic burden. Gross costing was used where micro-costing was not available (i.e. for any inpatient encounter outside of Calgary or Edmonton or any outpatient encounter). Gross costing is when aggregate resource use items are identified and expenditure data is collected at the organization level. Gross costs were identified from DAD and NACRS using resource intensity weights (RIW) for any healthcare encounter and were multiplied by the cost of a standard hospital stay (CSHS) in Alberta by year from the Canadian Institute for Health Information [14].

#### AHS corporate finance

Micro-costing data was available from AHS corporate finance for all inpatient admissions in Calgary and Edmonton. This is considered the gold standard of costing data. Patient level costs are provided and each component of resource use is estimated and a unit cost derived providing the most specific costing information possible [15]. This data includes the specific costs for each patient for nursing, operating room expenses, patient supplies, in-hospital drug use, allied healthcare, diagnostic imaging and testing (such as echocardiograms), laboratory testing, equipment costs (including equipment for specialized medical procedures such as hemodialysis), organization supports such as utilities, and housekeeping.

### Outcomes

The primary outcome was mean 12-month cumulative healthcare costs for all patients who had a CIED implant or generator replacement. Costs were also stratified into inpatient and outpatient costs. Additionally, healthcare utilization including number of inpatient and outpatient visits to any AHS facility were considered as well as LOS in hospital over 12 months. All outcomes were compared amongst those who did and did not develop a complex CIED SSI. All costs were inflated to 2022 Canadian dollars. The perspective taken was that of the pubic healthcare payer and therefore, patient-borne costs such as outpatient antibiotic prescriptions, were not included. Physician claims were not accessed for this work and thus were not included.

### Statistical analyses

Descriptive statistics, such as frequencies and percentages for categorical variables and means with standard deviation (SD) for continuous variables were used to describe baseline characteristics of patients with and without infections. For our primary method, we assessed the total mean costs, inpatient costs, and outpatient costs for all patients at one year after the index date of their implantation. Number of inpatient admissions, outpatient visits and total LOS were analyzed for all patients over the subsequent one year.

In order to adjust for covariates, a propensity score match was conducted for comparing CIED patients who developed a complex SSI within one year of implantation to those who did not develop an infection. Propensity scores were estimated using a logistic regression model with observed baseline characteristics of age, sex, Elixhauser comorbidity index [16] and device type (i.e., PM, ICD, CRT). Matching was performed using the greedy nearest-neighbor methods without replacement and a caliper of 0.2 of the standard deviation of the log-odds of the propensity score. To assess the balance in baseline characteristics, standardized mean differences in proportions between patients with and without complex SSIs for each covariate were calculated after matching. A weighted standardized difference < 10% indicated good balance and acceptable bias. Incremental healthcare utilization (i.e. costs, number of admissions/visits and LOS) were calculated to determine the effect of infection on these outcomes.

As a sensitivity analysis we conducted generalized linear models (GLM) to identify the relationship between the outcomes and infection. We used GLM with Gamma distribution and a log link function for the costs, GLM with Poisson distribution and a log link for number of hospital admissions, LOS, and number of outpatient visits.

All statistical analyses were performed using R Statistical Software (Version 1.4.0). This research was approved by the University of Calgary Health Research Ethics Board (REB20-2186).

## Results

### Patient characteristics

All patient characteristics are listed in Table 1. In total there were 26,049 index procedures identified from Paceart™ with 320 complex SSIs identified using administrative data. Females accounted for 36.2% of the devices implanted and 29.7% of the infections. The majority (70.4%) of patients had a PM implanted.

**Table 1:** Baseline characteristics of the cohort

Variables	Index procedures	Infection group	Rate of infection (%)
**All N**	26,049	320	1.23
**Age N (%)**			
18–29	340 (1.31)	16 (5)	4.71
30–39	474 (1.82)	11 (3.44)	2.32
40–49	901 (3.46)	20 (6.25)	2.22
50–59	2467 (9.47)	39 (12.19)	1.58
60–69	5200 (19.96)	76 (23.75)	1.46
70–79	7512 (28.84)	81 (25.31)	1.08
80+	9155 (35.15)	77 (24.06)	0.84
**Gender N (%)**			
F	9450 (36.28)	95 (29.69)	1.01
M	16,297 (62.56)	225 (70.31)	1.38
No Data	302 (1.16)	0 (0)	0
**Device Type N (%)**			
PM	18,329 (70.36)	182 (56.88)	0.99
CRT	3191 (12.25)	65 (20.31)	2.04
ICD	4375 (16.8)	70 (21.88)	1.6
LPM	73 (0.28)	0 (0)	0
S-ICD	80 (0.31)	3 (0.94)	3.75
No Data	1 (0)	0 (0)	0
**Generator Replacement N (%)**			
	6724 (25.81)	44 (13.75)	0.65
Pampalon: Material* N (%)			
1	4140 (15.89)	40 (12.5)	0.97
2	3917 (15.04)	48 (15)	1.23
3	4350 (16.7)	59 (18.44)	1.36
4	5041 (19.35)	64 (20)	1.27
5	5227 (20.07)	72 (22.5)	1.38
No Data	3374 (12.95)	37 (11.56)	1.1
Pampalon: Social** N (%)			
1	3457 (13.27)	48 (15)	1.39
2	2988 (11.47)	32 (10)	1.07
3	4402 (16.9)	68 (21.25)	1.54
4	5504 (21.13)	64 (20)	1.16
5	6324 (24.28)	71 (22.19)	1.12
No Data	3374 (12.95)	37 (11.56)	1.1
**Urban/Rural N (%)**			
Rural	4311 (16.55)	57 (17.81)	1.32
Urban	20,657 (79.3)	251 (78.44)	1.22
No Data	1081 (4.15)	12 (3.75)	1.11
**Number of Comorbidities N (%)**			
0–1	4740 (18.2)	13 (4.06)	0.27
2–3	9299 (35.7)	64 (20)	0.69
4–5	6386 (24.52)	78 (24.38)	1.22
6+	5624 (21.59)	165 (51.56)	2.93
**Elixhauser Index N (%)**			
-10-0	357 (1.37)	1 (0.31)	0.28
1–10	13,758 (52.82)	92 (28.75)	0.67
11–20	9029 (34.66)	129 (40.31)	1.43
21–30	2346 (9.01)	75 (23.44)	3.2
31–40	488 (1.87)	16 (5)	3.28
41–50	66 (0.25)	6 (1.88)	9.09
51–58	5 (0.02)	1 (0.31)	20

PM: Pacemaker, CRT: Cardiac Resynchronization Therapy, ICD: Implantable Cardioverter Defibrillator, LPM: Leadless Pacemaker; S-ICD: Subcutaneous Implantable Cardioverter Defibrillator

* Pampalon Deprivation Index - Material Indicators: Proportion of people aged 15 years and older with no high school diploma; Population employment ratio of people aged 15 years and older; Average income of people aged 15 years and older

**Pampalon Deprivation Index - Social Indicators: Proportion of individuals aged 15 years and older living alone; Proportion of individuals aged 15 years and older whose marital status is separated, divorced or widowed; Proportion of single-parent families

### Propensity score matching characteristics

Following propensity score matching, one infected patient did not have a match leading to a matched cohort of 319 (319 non-infection and 319 infection). Baseline characteristics between patients with and without infection were well balanced after matching on propensity score, as demonstrated by the standardized mean differences (Table 2).

**Table 2:** Baseline characteristics for propensity score matched pairs

	Infection (N = 319)	Non-Infection (N = 319)	Standardized Mean Difference
**Age, Mean (SD)**	67.29 (16.8)	66.64 (16.69)	0.04
**Sex, % (N)**			
Female	29.47 (94)	30.41 (97)	-0.02
Male	70.53 (225)	69.59 (222)	0.02
**Device Type, % (N)**			
PM	56.74 (181)	57.68 (184)	-0.02
CRT	20.38 (65)	18.81 (60)	0.04
ICD	21.94 (70)	21.94 (70)	0
S-ICD	0.94 (3)	1.57 (5)	-0.06
**Generator Replacement, % (N)**			
True	13.79 (44)	28.84 (92)	-0.37
**Elixhauser Index Score Groups, %(N)**			
-10-0	0.31 (1)	0.63 (2)	-0.05
1–10	28.84 (92)	28.53 (91)	0.01
11–20	40.44 (129)	41.69 (133)	-0.03
21–30	23.51 (75)	18.81 (60)	0.12
31–40	4.7 (15)	7.84 (25)	-0.13
41–50	1.88 (6)	2.19 (7)	-0.02
51–58	0.31 (1)	0.31 (1)	0

### Unadjusted costs

Patients with infection had substantially more total mean costs at one year $145,312 (Standard deviation (SD) 188,279) compared to those without infection, $34,264 (SD 53,790). These costs were largely driven by inpatient costs which accounted for 70% and 72% of costs in the non-infection and infection group, respectively (Table 3). Where microcosting data was available, the majority of inpatient costs were accounted for by nursing (36%) and organizational supports (15%).

**Table 3:** Costs for all patients

	Non-Infection	Infection
**Total Costs**		
N	25,729	320
Mean (SD)	34,264 (53,790)	145,312 (188,279)
**Inpatient Costs**		
Mean (SD)	23,988 (53,735)	130,456 (189,997)
**Outpatient Costs**		
Mean (SD)	10,276 (9,030)	14,766 (14,713)

### Adjusted costs

In the propensity score matched group, the mean incremental costs for the infection versus non-infection group was $94,128 (SD 190,185). Again, this was caused mainly by inpatient costs with a mean incremental difference of $90,620 (SD 193,833) compared to a mean incremental difference of $3,508 (SD 20,784) for the outpatient costs (Table 4). When the sensitivity test was done using GLM, the same relationships were demonstrated showing increased mean costs for the infection group overall (2.9 times greater than the non-infection group) and when stratified into inpatient and outpatient costs (3.9 and 1.3 times greater than the non-infection group, respectively).

Further sensitivity testing using GLM and including the variables of infection, device type, the five separate hospital centers of care for CIED implantations, and Pampalon deprivation index with an outcome of mean total 12 month costs, again demonstrated increased costs in the infection group. There was an additional $94,500 in costs for the infection group compared to non-infection group (*p* < 0.001) and incremental costs of $49,350 for those that had a CRT compared to PM (*p* < 0.005). There were no significant differences in costs when looking specifically at hospital center or Pampalon deprivation index classification.

**Table 4:** Costs for propensity-score matched pairs

	Non-Infection	Infection	Increment
**Total Costs**			
N	319	319	319
Mean (SD)	49757.39(73089.11)	143884.96(186833.61)	94127.57(190185.06)
**Inpatient Costs**			
Mean (SD)	38478.80(73820.52)	129098.74(188520.26)	90619.94(193832.86)
**Outpatient Costs**			
Mean (SD)	11278.59(11860.36)	14786.22(17184.63)	3507.63(20784.35)

### Number of outpatient visits, hospitalizations and LOS

Number of outpatient visits, hospitalizations, and LOS for all patients is shown in Table 5 and for the propensity score matched group in Table 6. For all patients, over a one-year time period, there was a mean number of admissions of 3.1 and 1.03 for those with and without infection, respectively. This contributed to LOS of 54.5 days in the infection group compared to 12.2 in the non-infection group. In the propensity score matched group there was an incremental increase of 1.6 admissions to hospital in the infected group compared to the non-infected group with an additional 34.8 days in hospital.

**Table 5:** Healthcare utilization for all patients

	Non-Infection	Infection
**Inpatient Admissions**		
N	25,729	320
Mean N (SD)	1.03(1.24)	3.10(1.94)
**Length of Stay**		
N	25,729	320
Mean days (SD)	12.24(29.61)	54.45(67.60)
**Outpatient Visits**		
N	25,729	320
Mean N (SD)	7.63(13.27)	16.26(23.93)

**Table 6:** Healthcare utilization for propensity score matched pairs

	Non-Infection	Infection	Increment
**Inpatient Admissions**			
N	319	319	319 pairs
Mean N (SD)	1.49 (1.67)	3.10 (1.94)	1.68 (2.27)
**Length of Stay**			
N	319	319	319 pairs
Mean N (SD)	18.92 (33.94)	53.69 (66.32)	34.77 (70.12)
**Outpatient Visits**			
N	319	319	319 pairs
Mean N (SD)	9.98 (15.99)	16.22 (23.96)	6.24 (29.72)

As an additional analysis, all costs and healthcare utilization including number of admissions and LOS were stratified by CIED device type. This information can be found in Additional File 1.

## Discussion

This work has demonstrated that infection following CIED implantation is related with substantial increased costs to the healthcare system compared to those who did not develop an infection. This was the case overall and when controlling for different variables using propensity score matching. The increase in costs was tied to greater healthcare utilization largely driven by inpatient hospitalizations with prolonged LOS.

The increase in hospitalizations and prolonged LOS is in keeping with the prior literature on complex SSI that occur after clean surgeries [17]. Complex SSIs frequently require intensive therapy to manage including repeat surgeries, prolonged courses of antimicrobials, and prolonged external pacing and monitoring in pacemaker-dependent patients or those with high risk life-threatening arrhythmias [18]. This results in substantial patient morbidity and prolonged LOSs [17]. Other work done specifically on CIED infections has also demonstrated significant increases in LOS compared to those who do not develop infection [9]. Prior work demonstrated increased costs associated with CIED infections, with incremental costs for a single admission ranging from $14,360 to $28,676 (in United States Dollars), depending on device type [2]. A prior retrospective analysis conducted in France explored costs associated with CIED implantation and generator replacement from 2012 to 2015 [10]. This work, similarly to ours and other existing literature, demonstrated that CIED infections were associated with increased cost particularly related to inpatient hospitalizations [10].

The increased economic burden associated with complex SSIs following CIED implantation, and the associated extensive healthcare utilization suggests a need for strategies to recognize and mitigate infection risk. Infection Prevention and Control (IPC) surveillance is one known strategy to monitor rates of infection and the impact of infection reduction strategy [19]. This type of surveillance has been recognized as a key area of importance for hospital systems and patient safety [19].

A strategy to reduce complex SSIs may include IPC interventions such as perioperative bundles including pre-operative checklists, decolonization for *Staphylococcus aureus* (a common causative pathogen for infection), appropriate preoperative antibiotics and hand hygiene [20]. A recent systematic review and meta-analysis demonstrated that these types of perioperative bundles are effective at reducing complex SSIs [21]. More novel solutions for reducing complex SSIs such as antibiotic impregnated envelopes inserted at the time of device implantation could also be considered to improve outcomes for patients, but are costly [5]. The work completed in this study can be used as part of the decision making process when implementing strategies to reduce complex SSIs. Understanding the economic burden is key to assessing value for money when choosing between different options to reduce infection.

As noted previously, the majority of costs associated with complex SSIs post-CIED implantation were found to be driven by inpatient hospitalizations with prolonged LOS. This is in keeping with previous literature demonstrating that prolonged hospitalizations are drivers of increased costs [22]. This may suggest a need for streamlining of services to manage complex SSIs in order to expedite discharge and reduce costs. A study done on orthopedic surgeries demonstrated that a dedicated infectious diseases team in conjunction with a joint arthroplasty service was able to successfully restructure patient discharge expeditiously while maintaining patient safety and quality of care [23].

This work is unique in that it used validated administrative data to track complex SSIs that occurred after CIED implantation [12]. This suggests increased reliability and validity of the results. There can be confidence in the ability of the data to identify infection and this work would be able to be replicated in other datasets. While costing data is specific to different regions and therefore can vary both in the national and international context, given that this work was population-based, and encompassed both rural and urban settings including academic and community settings, it is generalizable to different demographics and geographic areas. While costing studies should potentially be replicated in specific geographic locations, this work can provide a framework and foundation on which to replicate future studies. High quality micro-costing data was utilized where available improving the specificity of the costing estimates.

There are limitations that must be addressed. Infections were only tracked for one year, therefore complex SSIs occurring very late would not have been captured. There is always the possibility of errors in administrative data which relies on human coding. However, this provides one of the only ways to complete large population-based studies which would otherwise be prohibitively expensive and difficult. There are concerns associated with propensity score matching. One infection was excluded as a match could not be found; however, we do not believe that this introduced selection bias. There may be unobserved confounders that could influence the pairs and lead to bias in the matching, as all relevant differences may not be accounted for. Microcosting data was not available for all patients and given that many patients without infection were not re-admitted to hospital there would be more microcosting data available for the infection group. However, previous work on microcosting versus gross costing data specifically in Alberta exploring cardiac care demonstrated that microcosting and RIW performed similarly somewhat mitigating any risk of this discrepancy influencing costing outcomes [24]. Finally, physician claims were not included which may have influenced the costs from the public healthcare payer perspective. Given the increased hospitalizations and outpatient visits associated with the infection group, the physician costs would likely increase the differences even further.

## Conclusions

This work has demonstrated the substantial economic burden associated with CIED complex SSIs. Largely driven by hospital admissions and prolonged LOS, these costs demonstrate a need to mitigate infection risk and work toward strategies for prevention. Now that the foundation has been completed through assessment of economic burden, these findings can be applied to future studies exploring cost-effectiveness for different infection prevention strategies. This work would be of relevance to healthcare providers, guideline development committees and policy-makers. Further studies should be completed in larger populations and explore individual factors contributing to increased costs in complex CIED SSIs, such as individual comorbidities, comorbidity indices and causative pathogens.

### Electronic supplementary material

Below is the link to the electronic supplementary material.

Supplementary Material 1

## Data Availability

The data that support the findings of this study are available from Alberta Health Services but restrictions apply to the availability of these data, which were used under license for the current study, and so are not publicly available. Data are however available from the authors upon reasonable request and with permission of Alberta Health Services and appropriate ethics.

## Abbreviations

CIED: Cardiac Implantable Electronic Devices
PM: Pacemaker
ICD: Implantable Cardioverter Defibrillator
CRT: Cardiac Resynchronization Therapy
IPC: Infection Prevention and Control
SSI: Surgical Site Infection
AHS: Alberta Health Services
DAD: Discharge Abstract Database
NACRS: National Ambulatory Care Reporting System
ICD: International Classification of Diseases
LOS: Length of Stay
SD: Standard Deviation

## References

[1] Greenspon AJ, Patel JD, Lau E, Edmund L, Ochoa J, Frisch D (2011). 16-Year trends in the Infection burden for pacemakers and implantable cardioverter-defibrillators in the United States: 1993 to 2008. J Am Coll Cardiol.

[2] Sohail MR, Eby EL, Ryan MP, Gunnarsson C, Wright LA, Greenspon AJ (2016). Incidence, treatment intensity, and incremental annual expenditures for patients experiencing a cardiac implantable electronic device Infection. Circ: Arrhythm Electrophysiol.

[3] Canadian Institute for Health Information. Implantable medical devices in Canada: Insights into high-volume procedures and associated costs. https://secure.cihi.ca/free_products/implantable-medical-devices-report-en.pdf. Published 2020. Accessed June 15 2023.

[4] Dang T, Ngo L, Ali A, Hossain S, Kaambwa B (2019). Healthcare costs associated with early Complications following cardiovascular implantable electronic device implantation. Heart Lung Circ.

[5] Tarakji KG, Mittal S, Kennergren C, Corey R, Poole J, Stromberg K (2016). Worldwide randomized antibiotic envelope Infection prevention trial (WRAP-IT). Am Heart J.

[6] Rennert-May E, Chew D, Lu S, Chu A, Kuriachan V, Somayaji R (2020). Epidemiology of cardiac implantable electronic device Infections in the United States: a population-based cohort study. Heart Rhythm.

[7] Blomström-Lundqvist C, Traykov V, Erba PA, Burri H, Cosedis Nelson J, Grazia Bongiorni M (2020). European Heart Rhythm Association (EHRA) international consensus document on how to prevent, diagnose, and treat cardiac implantable electronic device infections-endorsed by the Heart Rhythm Society (HRS), the Asia Pacific Heart Rhythm Society (APHRS), the latin American Heart Rhythm Society (LAHRS), International Society for Cardiovascular Infectious Diseases (ISCVID) and the European Society of Clinical Microbiology and Infectious Diseases (ESCMID) in collaboration with the European Association for Cardio-Thoracic Surgery (EACTS). Europace.

[8] Daneman N, Homenauth E, Saskin R, Ng R, Ha A, Wijeysundera HC (2020). The predictors and economic burden of early-, mid- and late-onset cardiac implantable electronic device Infections: a retrospective cohort study in Ontario, Canada. Clin Microbiol Infect.

[9] Sohail MR, Henrikson CA, Braid-Forbes MJ, Forbes KF, Lerner DJ (2011). Mortality and cost associated with cardiovascular implantable electronic device Infections. Arch Intern Med.

[10] Clémenty N, Carion PL, Léotoing Ld LL, Wilquin-Bequet F, Brown B (2018). Infections and associated costs following cardiovascular implantable electronic device implantations: a nationwide cohort study. Europace.

[11] Eby EL, Bengtson LGS, Johnson MP, Burton ML, Hinnenthal J (2020). Economic impact of cardiac implantable electronic device Infections: cost analysis at one year in a large U.S. health insurer. J Med Econ.

[12] Rennert-May E, Leal J, MacDonald M, Cannon K, Smith S, Exner D (2022). Validating administrative data to identify surgical site Infections following cardiac implantable electronic device implantation: a comparison of traditional methods and machine learning. Antimicrob Resist Infect Control.

[13] Pampalon R, Hamel D, Gamache P, Raymond G (2009). A deprivation index for health planning in Canada. Chronic Dis Can.

[14] Pink GH, Bolley HB (1994). Physicians in health care management: 3. Case Mix groups and Resource Intensity weights: an overview for physicians. CMAJ.

[15] Xu X, Lazar CM, Ruger JP (2021). Micro-costing in health and medicine: a critical appraisal. Health Econ Rev.

[16] Sharma N, Schwendimann R, Endrich O, Ausserhofer D, Simon M (2021). Comparing Charlson and Elixhauser comorbidity indices with different weightings to predict in-hospital mortality: an analysis of national inpatient data. BMC Health Serv Res.

[17] Rennert-May ED, Conly J, Smith S (2018). The cost of managing complex surgical site Infections following primary hip and knee arthroplasty: a population-based cohort study in Alberta, Canada. Infect Control Hosp Epidemiol.

[18] Rastan AJ, Doll N, Walther T, Mohr FW (2005). Pacemaker dependent patients with device infection—a modified approach. Eur J Cardiothoracic Surg.

[19] Storr J, Twyman A, Zingg W, Damani D, Kilpatrick C, Reilly J (2017). Core components for effective Infection prevention and control programmes: new WHO evidence-based recommendations. Antimicrob Resist Infect Control.

[20] Sastry S, Rahman R, Yassin MH (2015). Cardiac implantable electronic device Infection: from an Infection prevention perspective. Adv Prev Med.

[21] Wolfhagen N, Boldingh QJJ, Boermeester MA, de Jonge SW (2022). Perioperative care bundles for the prevention of surgical-site Infections: meta-analysis. Br J Surg.

[22] Badia JM, Casey AL, Petrosillo N, Hudson PM, Mitchell SA, Crosby C (2017). Impact of surgical site Infection on healthcare costs and patient outcomes: a systematic review in six European countries. J Hosp Infect.

[23] Goodson KM, Kee JR, Edwards PK, Novack A, Stambough JB, Siegel E (2020). Streamlining hospital treatment of prosthetic joint Infection. J Arthroplasty.

[24] Clement F, Ghali W, Donaldson C, Manns B (2009). The impact of using different costing methods on the results of an economic evaluation of cardiac care: microcosting vs gross-costing approaches. Health Econ.

