# Supplementary material for: The economic burden of cardiac implantable electronic device infections in Alberta, Canada: a population-based study using validated administrative data
Source: Antimicrob Resist Infect Control. 2023 Dec 5;12:140. doi: 10.1186/s13756-023-01347-4 (PMC10698885; doi:10.1186/s13756-023-01347-4)
Supplement: Supplementary file 1 — Supplementary Material 1 [file 13756_2023_1347_MOESM1_ESM.docx]

**Additional File 1**

Table 1. Healthcare utilization stratified by device type for the non-infection group

| **Non-Infection Group** | **ALL**  **(N=**25729**)** | **PM**  **(N=**18147**)** | **CRT**  **(N=**3126**)** | **ICD**  **(N=**4305**)** | **LPM**  **(N=**73**)** | **S-ICD**  **(N=**77**)** | **NA**  **(N=1)** |
| --- | --- | --- | --- | --- | --- | --- | --- |
| **Total Cost** | | | | | | | |
| Mean (SD) | 34263.8 (53790.06) | 31343.83 (49850.75) | 42333.99 (64995.3) | 40261.29 (59258.54) | 36155.53 (50593.71) | 57693.71 (61881.76) | 34214.52 (NA) |
| Median (IQR) | 19299.86 (24580.22) | 16793.11 (19480.96) | 30072.57 (26449.34) | 28946.1 (28810.67) | 16082.68 (28924.35) | 38808.93 (23994.37) | 34214.52 (0) |
| **Inpatient Cost** | | | | | | | |
| Mean (SD) | 23988.26 (53735.73) | 22664.19 (49743.89) | 26631.05 (65742.52) | 27438.26 (59760.22) | 20023.69 (39862.14) | 39540.43 (59876.14) | 30311.49 (NA) |
| Median (IQR) | 11168.51 (26950.04) | 11527.95 (21736.8) | 6413.65 (32149.21) | 12371.31 (32914.81) | 9945.13 (18551.75) | 28033.3 (38516.17) | 30311.49 (0) |
| **Outpatient Cost** | | | | | | | |
| Mean (SD) | 10275.54 (10574.75) | 8679.64 (8462.89) | 15702.95 (13721.13) | 12823.03 (12842.1) | 16131.84 (31512.7) | 18153.28 (21213.67) | 3903.03 (NA) |
| Median (IQR) | 8520.39 (10383.61) | 8210.17 (9438.27) | 9849.85 (23372.43) | 8934.6 (17466.37) | 5903.52 (9686.85) | 8100.53 (31305.38) | 3903.03 (0) |
| **Index Costs (Costs for Encounter Associated with Index Date)** | | | | | | | |
| Mean (SD) | 19907.99 (33981.38) | 16954.73 (31621.07) | 25977.91 (34001.3) | 27763.66 (41177.74) | 10643.64 (13310.19) | 38939.54 (29949.37) | 30311.49 (NA) |
| Median (IQR) | 13290.18 (13063.85) | 11303.93 (7735.37) | 27034.53 (22040.57) | 26050.57 (23965.34) | 8468.17 (14009.91) | 32471.96 (20992.84) | 30311.49 (0) |
| **Follow Up Costs (Costs for Subsequent Encounters after Index Encounter)** | | | | | | | |
| Mean (SD) | 14355.81 (38608.87) | 14389.1 (35472.22) | 16356.08 (53308.17) | 12497.63 (38021.16) | 25511.89 (47140.74) | 18754.18 (53571.02) | 3903.03 (NA) |
| Median (IQR) | 2217.71 (11420.67) | 2186.34 (11902.62) | 2828.16 (12401.47) | 1974.12 (8912.06) | 3612.88 (21385.45) | 810.59 (4969.9) | 3903.03 (0) |
| **Inpatient admits** | | | | | | | |
| Mean (SD) | 1.03 (1.24) | 1.05 (1.24) | 0.97 (1.3) | 0.97 (1.22) | 1.04 (1.07) | 1.29 (1.12) | 1 (NA) |
| Median (IQR) | 1 (1) | 1 (1) | 1 (1) | 1 (1) | 1 (2) | 1 (0) | 1 (0) |
| **Inpatient LOS** | | | | | | | |
| Mean (SD) | 12.24 (29.61) | 13.14 (31.49) | 10.41 (26.74) | 9.85 (22.56) | 12.53 (30.15) | 9.53 (14.4) | 2 (NA) |
| Median (IQR) | 3 (11) | 3 (11) | 2 (9) | 2 (10) | 2 (7) | 2 (12) | 2 (0) |
| **Outpatient Visits** | | | | | | | |
| Mean (SD) | 7.63 (13.27) | 7.03 (12.25) | 8.27 (12.63) | 9.22 (15.43) | 27.23 (48.96) | 15.14 (29.1) | 15 (NA) |
| Median (IQR) | 4 (6) | 4 (6) | 4 (8) | 4 (8) | 10 (12) | 3 (15) | 15 (0) |

Table 2. Healthcare utilization stratified by device type for the infection group

| **Healthcare Utilization** | **ALL**  **(N=**320**)** | **PM**  **(N=**182**)** | **CRT**  **(N=**65**)** | **ICD**  **(N=**70**)** | **S-ICD**  **(N=3)** |
| --- | --- | --- | --- | --- | --- |
| **Total Cost** | | | | | |
| Mean (SD) | 145312.09 (188279.35) | 131109.06 (177025.21) | 166598.25 (226950.69) | 162848.5 (179966.78) | 136579.31 (92149.64) |
| Median (IQR) | 88121.96 (97130.82) | 78693.24 (96138.26) | 103634.98 (105028.69) | 105591.24 (115314.26) | 98837.72 (86158.21) |
| **Inpatient Cost** | | | | | |
| Mean (SD) | 130546.17 (189997.11) | 119107.17 (177306.62) | 147773.36 (230119.73) | 146133.11 (184475.38) | 87562.03 (105843.43) |
| Median (IQR) | 72652.94 (99359.41) | 66984.54 (98338.94) | 93990.67 (123775.98) | 74677.44 (115432.76) | 44910.88 (99189.17) |
| **Outpatient Cost** | | | | | |
| Mean (SD) | 14765.91 (17161.52) | 12001.89 (15557.37) | 18824.9 (15355.64) | 16715.4 (19541.54) | 49017.27 (35046.61) |
| Median (IQR) | 10482.19 (13878.84) | 9453.55 (11289.48) | 14351.25 (22076.76) | 10482.19 (22143.18) | 33531.53 (32379.14) |
| **Index Costs (Costs for Encounter Associated with Index Date)** | | | | | |
| Mean (SD) | 39429.83 (79072.86) | 32838.28 (58996.49) | 32809.45 (44695.32) | 62003.65 (130752.67) | 56036.28 (70070.78) |
| Median (IQR) | 17590.97 (26508.28) | 14093.32 (18049) | 27478.57 (28121.06) | 31885.9 (30810.9) | 33260.93 (67237.47) |
| **Follow Up Costs (Costs for Subsequent Encounters after Index Encounter)** | | | | | |
| Mean (SD) | 105882.26 (172006.87) | 98270.77 (165590.8) | 133788.8 (221188.74) | 100844.86 (136088.72) | 80543.03 (22934.37) |
| Median (IQR) | 58522.53 (84402.27) | 48652.29 (74194.69) | 73888.19 (92565.16) | 59976.6 (76028.83) | 69105.41 (20685.04) |
| **Inpatient admits** | | | | | |
| Mean (SD) | 3.1 (1.94) | 2.76 (1.52) | 3.57 (2.29) | 3.56 (2.39) | 3 (1.73) |
| Median (IQR) | 3 (2) | 2.5 (1) | 3 (3) | 3 (3) | 2 (1.5) |
| **Inpatient LOS** | | | | | |
| Mean (SD) | 54.45 (67.6) | 52.06 (55.61) | 65.45 (104.72) | 51.41 (51.53) | 32.33 (29.87) |
| Median (IQR) | 35.5 (48) | 31 (44.75) | 37 (58) | 37 (45.5) | 22 (28.5) |
| **Outpatient Visits** | | | | | |
| Mean (SD) | 16.26 (23.93) | 14.22 (23.11) | 19.98 (23.84) | 17.06 (23.69) | 41 (60.75) |
| Median (IQR) | 8 (17) | 7 (14) | 9 (24) | 9 (17) | 10 (54.5) |
